# Editorial: Insights in dental materials: 2022

**DOI:** 10.3389/fdmed.2023.1195007

**Published:** 2023-04-20

**Authors:** Josette Camilleri

**Affiliations:** School of Dentistry, Institute of Clinical Sciences, College of Medical and Dental Sciences, University of Birmingham, Birmingham, United Kingdom

**Keywords:** material insights, caries management, bonding systems, light curing, bulk fill composites, mechanical testing, material informatics, medical device regulation

**Editorial on the Research Topic** Insights in dental materials: 2022

The Research Topic *Insights in Dental Materials: 2022* was put together to bring to the reader key innovations and opinions in the field of dental materials. I have led this Research Topic personally as I felt it was a crucial time for the journal to have such a collection where the top opinion leaders share their knowledge and advancements in the field.

This Research Topic includes three reviews, two mini reviews and a method article. The insights includes 3 clinical-focused articles that discuss the scientific basis of clinical protocols for the management of the carious tooth, bonding systems and understanding light curing and effective ways of ensuring full curing of deep bulk fill composites. The use of material informatics to develop smarter materials and how to effectively test the physical properties follow with in depth knowledge of the medical device directive and how it effects the development and marketing of the materials we use in clinical practice.

Dental caries is still highly prevalent world-wide and despite the advances in materials and clinical techniques there are still a lot of controversies on the best practice for the management of dental caries. This is addressed in *The scientific management of deep carious lesions in vital teeth using contemporary materials—A narrative review* by Al-Ali and Camilleri. The narrative review discusses the contrasting position statements on the management of deep caries and the exposed dental pulp issued by the European Society of Endodontology and the American Association of Endodontists. These statements were analysed together with the original scientific research and clinical articles and guidance for clinicians has been suggested for the best practice in managing deep carious lesions.

Bonding system are also discussed in *Insight into the development of versatile dentin bonding agents to increase the durability of the bonding interface* by Celerino de Moraes Porto et al. highlighting the improvements in bonding technology over the past 50 years. Regardless of the technological improvements hydrolytic degradation, combined with the action of dentin matrix enzymes, destabilizes the tooth-adhesive bond and leads to long term degradation and failure. The review looks at how to inhibit metalloproteinase action by using antimicrobial adhesive systems and also by preparation of the dentine to reduce collagen degradation. This insight enhances the understanding of dentine bonding systems and opens a lot of insight into further material development in this field.

The importance of adequate light curing of composite resins is stressed in the article by Watts, *Light-curing dental resin-based composites: How it works and how you can make it work*. With the restriction in the use of dental amalgam, all plastic restorative materials are light cured. Scientific knowledge of how the light beam interacts with the material is important as inadequate light curing of composite resin restorations will result in defective restorations prone to secondary caries. This article is essential for clinicians to avoid insufficient light curing of bulk fill composites and enhances the clinician's knowledge of materials.

In *Materials informatics for developing new restorative dental materials: A narrative review* by Yamaguchi et al*.*, the authors review the advantages, limitations, and future perspectives of materials informatics over a span of 20 years exploring the optimum compositions in developing new materials using artificial intelligence. This is done by mapping crucial material properties for each material type. Then from the material informatics point of view the data is prepared with descriptors denoted as x and known values from *in vitro* studies listed as y. A regression model is then developed and if this is good enough the optimum descriptors that achieve desirable material properties can be inversely searched. Using materials informatics will accelerate the process of restorative materials development and contribute to produce new insights into dental materials research.

Another review by Darvell, entitled *Mechanical test relevance—A personal perspective on some methods and requirements,* questions the appropriateness of mechanical tests that are undertaken to test dental materials. This article discusses in detail how the test is selected and whether the selected test is appropriate to define what is being measured which is at times not possible, so a proxy method needs to be selected. The different mechanical tests are described in detail with the advantages and limitations of each test to guide the operator on test selection and interpretation.

The review article *The medical device regulation (MDR) from a dental perspective* by Mohn and Zehnder explains the new medical device regulation (EU 2017/745) adopted by the European Union in 2017 which has broad effects on manufacturers, importers, distributors, users of medical devices, and patients. This regulation will create more paperwork for dentists and red tape for the manufacturers and distributors with some companies being inappropriately disadvantaged. However, this regulation can also create new opportunities for researchers in dental materials. It is still unclear whether the medical device regulation will be of any benefit to patients and whether it will achieve the aim of having less products which are better regulated.

These articles which are published in *Insights in Dental Materials: 2022* show the limitations of the materials we currently use for management of dental disease and also provide insight on better ways of understanding the materials and improve the clinical protocols used to optimize material properties. The *in vitro* testing is challenging and may not be clinically translatable. Artificial Intelligence is promising and will help the development of better and smarter materials which hopefully can get the certification for use in practice. I would like to extend my sincere gratitude to all authors who have contributed to make this Research Topic a success and for sharing their knowledge.

